# Respiratory syncytial virus: can we still believe that after pandemic bronchiolitis is not a critical issue for public health?

**DOI:** 10.1007/s00431-023-05201-y

**Published:** 2023-09-20

**Authors:** Anna Chiara Vittucci, Livia Antilici, Cristina Russo, Anna Maria Caterina Musolino, Sebastian Cristaldi, Renato Cutrera, Sabrina Persia, Chiara Velia Di Maio, Massimiliano Raponi, Carlo Federico Perno, Alberto Villani

**Affiliations:** 1 Emergency, Acceptance and General Pediatrics Department, Bambino Gesù Children’s Hospital IRCCS, Piazza Sant’Onofrio 4-00165, Rome, Italy; 2 Microbiology and Diagnostic Immunology Unit, Bambino Gesù Childrens Hospital, IRCCS, Rome, Italy; 3 Pediatric Pulmonology and Cystic Fibrosis Unit, Bambino Gesù Children’s Hospital IRCCS, Rome, Italy; 4 Medical Direction, Bambino Gesù Childrens Hospital, IRCCS, Rome, Italy; 5 System Medicine Department, Tor Vergata University of Rome, Rome, Italy

**Keywords:** RSV, Bronchiolitis, Infants, SarsCOV2, Prophylaxis, Vaccine

## Abstract

Respiratory syncytial virus (RSV) is the leading cause of lower respiratory tract infection among infants and young children, resulting in annual epidemics worldwide. Since the beginning of the COVID-19 pandemic, non-pharmacological interventions were applied, interfering with the circulation of most respiratory viruses, including RSV. The aim of this study is to analyze the RSV infection trend among hospitalized infants during the actual epidemic season (2022–2023) in comparison with the last pre-pandemic season (2018–2019), in order to outline whether significant differences emerge due to COVID-19 pandemia. We retrospectively reviewed medical data on infants hospitalized at the Bambino Gesù Children’s Hospital with diagnosis of bronchiolitis in the current epidemic season and in the last pre-pandemic season, 2018–2019. RSV remains the main etiological agent of bronchiolitis in terms of frequency and severity of infections in the ongoing epidemic season. The first RSV case of the 2022–2023 season was detected at week 42 *vs* week 47 in the 2018–2019 season. The length of epidemic season was of 17 weeks in 2022–2023 *vs* 18 weeks in 2018–2019. Comparing the two seasons, age at admission was significantly higher in the current season (median age 2022–2023 65 days *vs* median age 2018–2019 58 days), but the disease severity was similar.

*       Conclusions*: The 2022–2023 bronchiolitis season in Italy started earlier than the usual pre-pandemic seasons but seasonality pattern may be going back to the pre-pandemic one. This season was not more severe than the previous ones. The impact of RSV disease on health care systems and costs remains a critical issue.

**Table:** 

**What is Known:** *• RSV is one of the major leading causes of hospitalization among children aged less than 3 months. SarsCOV2 pandemic interfered with the seasonal circulation of most respiratory viruses, Including RSV.*	
**What is New:** *• The 2022–2023 bronchiolitis season in Italy started and peaked earlier than the usual pre-pandemic seasons but seasonality pattern may be realigning to the pre-pandemic one. The impact of RSV disease on health care systems and costs is concerning.*

**What is Known:**

*• RSV is one of the major leading causes of hospitalization among children aged less than 3 months. SarsCOV2 pandemic interfered with the seasonal circulation of most respiratory viruses, Including RSV.*

**What is New:**

*• The 2022–2023 bronchiolitis season in Italy started and peaked earlier than the usual pre-pandemic seasons but seasonality pattern may be realigning to the pre-pandemic one. The impact of RSV disease on health care systems and costs is concerning.*

## Introduction

Bronchiolitis is the major cause of hospital admission in children aged less than 12 months in developed countries [1]. Respiratory syncytial virus (RSV) is the most common causative agent, with the highest burden in infants younger than 3 months [2]. A birth cohort study carried out in five European countries between 2017 and 2021 found that 1.8% of term-born children are admitted to the hospital due to RSV infection in the first year of life [3]. It is estimated that more than 90% of children are infected by the age of 2 years [4, 5].

RSV is classified into two major antigenic and genetic groups (RSV A and B) based on the characterization of the G protein gene; in addition, multiple genotypes have been identified within each of these groups. The 2 subtypes co-circulate during the same season, but in general, one predominates [6].

Clinical manifestations may range from mild disease with spontaneous resolution to severe infections which requires hospitalization and oxygen support and may eventually lead to pediatric intensive care unit (PICU) admission [1].

This disease has onset, offset, duration, and peak characteristics that may slightly vary each year. Seasonality also varies according to the geographical hemisphere; in the northern hemisphere, the season usually lasts from October to April, peaking between December and February, contrary to the southern hemisphere, where it may start as early as July [7, 8], establishing the beginning of the annual season. There are well-defined risk factors for severe RSV, including age, prematurity, chronic lung diseases, congenital heart disease, or immunodeficiency [1, 9]. Despite the greater risk of hospitalization due to RSV among children with these conditions, full term infants with no underlying illness account for a substantially higher absolute number of RSV hospitalizations [10].

There are still no vaccines available and current treatment strategies are limited to supportive care. The only available preventive strategy is prophylaxis with a monoclonal antibody (mAb), palivizumab, which requires monthly administration throughout the RSV season, and its use is restricted to high-risk infants [11].

Since December 2019, SARS-CoV-2 started circulating and rapidly led to a world pandemic. Non-pharmacological interventions, including protective masks and restriction orders, were applied based on national decisions, interfering with the circulation of most respiratory viruses, including RSV [12]. During the first weeks of restrictions applied in Italy because of the pandemic (March 2020), a relevant decrease in the number of attendances to pediatric emergency departments was observed, compared with the same period in 2019 [13]. Concomitantly, a drastic reduction in circulation of respiratory viruses, including RSV in the 2020–2021 season, were observed in both the northern and southern hemispheres. The incidence of bronchiolitis and, consequently, hospitalizations and PICU admissions decreased [14, 15].

However, the relaxation of public health measures resulted in a return of bronchiolitis disease burden in infants and in the appearance of peaks in atypical RSV seasons both in southern and northern hemispheres [16–20].

The 2021–2022 bronchiolitis season in Italy started and peaked earlier than the usual pre-pandemic seasons but had a shorter duration [21]. Although RSV peaks showed atypical patterns in 2021 and 2022, no increase in disease severity was reported [22].

The aim of this study is to analyze bronchiolitis hospitalization and RSV infection’s trend among infants during the actual epidemic season, 2022–2023. As a secondary objective, we aim to compare the ongoing epidemic season with the last pre-pandemic season (2018–2019), in order to assess whether significant differences emerge due to the COVID-19 pandemic onset.

## Material and methods

We retrospectively reviewed the medical records of children hospitalized with diagnosis of bronchiolitis at the Emergency and General Pediatric Unit of Bambino Gesù Children’s Hospital, Rome, Italy, between October 1st and February 28th of the current epidemic season 2022–2023. Additionally, we collected data from the same period of the last pre-pandemic season, 2018–2019. Bronchiolitis was a clinical diagnosis, based on anamnestic report and physical examination (infants presenting with their first episode of lower respiratory infection, who had diffuse crackles on auscultation). Only children with less than 1 year of age were enrolled in the study. We included only infants hospitalized with a well-characterized definition of bronchiolitis. Data were extracted from medical records, included age, sex, perinatal history, siblings, breastfeeding, and length of hospitalization (LOS), in addition to data regarding length of oxygen therapy and modality of respiratory support: standard oxygen therapy (SOT), high-flow nasal cannulas (HFNC), helmet continuous positive airway pressure (HcPAP), and invasive mechanic ventilation (IVM). We excluded patients who had previously received a diagnosis of bronchiolitis and those requiring prolonged hospitalization for concomitant diseases not related to bronchiolitis. A nasal swab performed at admission was used to identify the causative agents. Samples collected were processed, immediately or after storage at − 80 °C. The identification of respiratory viruses was accomplished by the multiplex RTPCR “Allplex^TM^ Respiratory Panel Assays” on All-in-One Platform (Seegene, Republic of Korea). Nucleic acids were extracted using the STARMag Universal Cartridge Kit (Seegene, Republic of Korea) on the automated Nimbus IV platform that can process 30 samples per run. Two hundred µL of sample was extracted, and nucleic acid was eluted with 100 µL of elution buffer. Real time PCR was performed on CFX96 (Bio Rad Laboratories); for each reaction, 8 µL of the extracted DNA/RNA in a final volume of 25 µL were used. The panel is composed of 3 multiplex PCR for the detection of 16 different viruses (Influenza A and B virus, Respiratory syncytial virus A and B, Adenovirus, Enterovirus, Parainfluenza virus 1, 2, 3, and 4, Metapneumovirus, Bocavirus, Rhinovirus, and 3 Coronaviruses NL63/229E/OC43). An internal control was included in each sample to check both extraction efficiency and PCR inhibition. In every run, a negative control was used to monitor the carry-over contamination and a positive control to check PCR reaction. The results were analyzed automatically using the Seegene software (Seegene Viewer V2.0, Seoul, Korea). According to datasheet indications for result interpretation, samples with a cycle threshold (Ct) ≤ 42 were considered positive; samples with no Ct or a Ct > 42 were considered negative. The whole workflow was performed as recommended by the manufacturer. In addition, a nasopharyngeal swab for the identification of SARS-CoV-2 was carried out from all children admitted after 2020. Starting from 2020, SARS-CoV-2 molecular assay or SARS-CoV-2antigen tests were performed.

In accordance with previously published works, RSV epidemic season was defined as the weeks when RSV detection exceeded 1.2% of total RSV specimen [8]. In addition, RSV detections had to constantly exceed this threshold during the season, with only a 1-week gap allowed. The end of epidemic season was calculated with the same method. We calculated the peaks and median length of the season based on the season-specific epidemic threshold. The season peak was defined as the week in which the highest number of RSV was isolated. If 2 weeks had the same number of detections, the first week was defined as the peak week.

We also investigated if patients with viral coinfections had different demographic and clinical characteristics compared to those with a single etiological agent detected.

The study protocol was approved by the Institutional Review Board and Ethics Committee of our institution (Protocol 2053-OPBG). The study was performed in line with the principles of the Declaration of Helsinki and its later amendments or comparable ethical standards.

### Statistical analysis

Categorical variables are presented as number and percentages, and distributions of categorical data were compared with either Pearson’s *χ*^2^ test or Fisher’s exact test, as appropriate. Continuous variables are presented as medians with corresponding interquartile ranges (IQRs): direct comparisons were made with Mann–Whitney *U* tests or Kruskal–Wallis tests. To evaluate the relationship between the variables, Spearman’s correlation analysis was performed.

Statistical analysis was performed using the software SPSS 26.0 version.

## Results

### 2022–2023 season

A total of 294 children with a diagnosis of bronchiolitis were included in our analysis. No significant gender difference was noted (45.2% female, 54.8% male) in the sample size. Median age at admission was 65.10 days (interquartile range, IQR 43.40–122.55 days); median LOS was 4.9 days (IQR 3.0–6.9 days). 81.3% of patients required oxygen therapy, 17.7% needed HcPAP and 2.7% needed mechanical ventilation. No children died. Most of our patients (83.7%) had neither a story of prematurity nor comorbidities. In fact, in our cohort, 35 patients were born preterm and 8 of them received palivizumab. Moreover, only 5.4% of patients (16) had comorbidities: 6 had congenital heart disease, 1 chronic lung disease of prematurity, and 9 other miscellaneous diseases. Demographic and clinical characteristic are summarized in Table 1. Nasal swabs revealed RSV as the main etiological agent (69.7%), followed by rhinovirus (22.4%), influenza (7.8%), and parainfluenza (6.1%). Viral coinfection was detected in 24.1% of patients.

**Table 1:** Clinical and demographic characteristics of inpatients of pre-pandemic and post-pandemic seasons

	**2018–2019** **(*N* = 300)**	**2022–2023** **(*n* = 294)**	***P*-value**
**Male, *N* (%)**	146 (48.7)	161 (54.8)	0.137
**Age, Days (IQR)**	58.2 (37.5–100.6)	65.1 (43.4–122.5)	0.038
**Preterm Birth, *N* (%)**	33 (11.2)	35 (11.7)	0.867
**Palivizumab, *N* (%)**	4 (1.3)	8 (2.7)	0.256
**Birth Weight, Grams (IQR)**	3200 (2900–3550)	3320 (2960–3580)	0.086
**Breastfeeding, *N* (%)**	228 (76)	227 (77.2)	0.727
**Siblings, *N* (%)**	159 (76.4)	190 (68.3)	0.049
**Comorbidities**	12 (4.0)	16 (5.5)	0.397
**Los, days (IQR)**	5.9 (3.9–7.9)	4.9 (3.0–6.9)	< 0.001
**Need For O**_**2**_**, *N* (%)**	206 (68.7)	239 (81.3)	< 0.001
**LOO, Days (IQR)**	2.75 (0–5)	3 (1–5)	0.032
**Need for SOT, *N* (%)**	110 (36.7)	136 (46.3)	0.018
**SOT, Days (IQR)**	2 (1.25–3)	2 (1–3)	0.199
**Need for HFNC, *N* (%)**	121 (40.3)	140 (47.6)	0.074
**HFNC, Days (IQR)**	4 (3–5)	3 (2–5)	0.067
**Need for HCPAP, *N* (%)**	46 (15.3)	52 (17.7)	0.440
**HCPAP, Days (IQR)**	3 (2–5)	3 (2–4)	0.132
**Need for IMV, *N* (%)**	11 (3.7)	8 (2.7)	0.513
**IMV, Days (IQR)**	5 (4.5–8)	4 (4–6)	0.012
**RSV+ **	231 (77.0)	205 (71.2)	0.107
**Los, Days (IQR)**	5.9 (4.9–8.9)	4.9 (3.9–6.9)	< 0.001
**Need for O**_**2**_**, *N* (%)**	179 (77.5)	186 (90.1)	< 0.001
**Need for SOT, *N* (%)**	100 (43.3)	106 (51.7)	0.079
**Need for HFNC, *N* (%)**	104 (45.0)	106 (51.7)	0.163
**Need for HcPAP, *N* (%)**	45 (19.5)	44 (21.5)	0.608
**Need for IMV, *N* (%)**	11 (4.8)	6 (2.9)	0.323

**Fig. 1:**
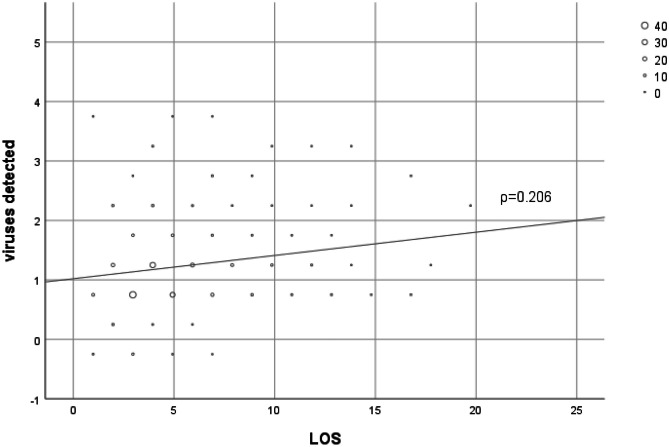
Correlation between number of viral detection and LOS (in days)

Comparing patients with single infection vs those with co-infections, the age of patients in the coinfection group was significantly higher compared to the single-agent group (single agent 60.2 days IQR 40.4–99.6 *vs* coinfections 77.9 days IQR 51.3–179.5; *p* = 0.005). Overall, no differences emerged in terms of LOS (single agent 4.9 days IQR 2.9–6.9 *vs* coinfections 4.9 days IQR 2.9–7.1; *p* = 0.17), need for O_2_ (single agent 84% *vs* coinfections 83.1%; *p* = 0.86), and need for HcPAP in the two groups (single agent 18% *vs* coinfections 21.1%; *p* = 0,56). Anyway, the number of viral agents detected positively correlates with days of oxygen therapy (*ρ* = 0.129 *p* = 0.027), age (*ρ* = 0.143 *p* = 0.014), and length of stay (*ρ* = 0.206 *p* =  < 0.001) (Fig. 1).

In addition, we divided patients into RSV+ group and in RSV − group. RSV+ patients showed a significantly more severe infection than RSV− patients: RSV+ bronchiolitis required longer hospitalization, needed more frequently oxygen-therapy, and differed significantly in terms of length of oxygen supplementation (LOO) (Table 2).

**Table 2:** Demographic, clinical characteristics, and disease severity according to main etiological group in the epidemic season 2022–2023

	**RSV+ ** **(*N* = 205)**	**RSV–** **(*N* = 83)**	***P*-value**
**Male, *N* (%)**	105 (51.2)	50 (60.2)	0.164
**Age, Days (IQR)**	65.1 (43.4–116.4)	63.1 (42.9–133.6)	0.927
**Preterm Birth, *N* (%)**	23 (11.2)	9 (10.8)	0.926
**Palivizumab, *N* (%)**	3 (1.5)	5 (6.0)	0.033
**Birth weight, grams (IQR)**	3330 (2900–3580)	3300 (3040–3555)	0.933
**Breastfeeding, *N* (%)**	158 (77.4)	64 (81.0)	0.939
**Siblings, *N* (%)**	132 (68.4)	55 (68.7)	0.954
**Comorbidities**	8 (3.9)	8 (9.8)	0.052
**Los, Days (IQR)**	4.9 (3.9–6.9)	2.9 (2.9–4.9)	< 0.001
**Need for O**_**2**_**, *N* (%)**	186 (90.7)	51 (61.44)	< 0.001
**LOO, Days (IQR)**	4 (2–6)	2 (0–4)	< 0.001
**Need for SOT, *N* (%)**	106 (78.5)	29 (21.5)	0.010
**SOT, Days (IQR)**	2.0 (1–3.2)	2.0 (1.0–2.0)	0.257
**Need for HFNC, *N* (%)**	106 (51.7)	33 (39.7)	0.066
**HFNC, Days (IQR)**	3 (2.0–4.5)	3 (2.0–5.0)	0.579
**Need for HcPAP, *N* (%)**	44 (21.5)	8 (15.4)	0.018
**HcPAP, Days (IQR)**	3 (2.25–4)	3 (1.25–3.75)	0.316
**Need for IMV, *N* (%)**	6 (2.9)	2 (2.4)	0.809
**IMV, Days (IQR)**	5 (3.75–6.25)	4 (4.0–4.0)	0.643

Analyzing the RSV+ group, we subdivided patients into two further subgroups: children with single RSV infection and children with RSV+ plus other viral agents. No statistical difference emerged in terms of severity of infection (data not shown).

In the 2022–2023 season, RSV-B was the primary circulating subtype, accounting for 85% of the subtyped samples (68/80). Only sporadic cases of RSV-A (12/80) were observed.

Moreover, we divided all our patients into two groups, based on severity of infection: patients requiring SOT and HFNC *vs* patients requiring a stronger ventilatory support such as HcPAP and/or IMV (Table 3). Not surprisingly, the second group required prolonged hospitalization and showed higher oxygen need. Coinfection rate did not differ between the two groups, while RSV was more frequent in the HcPAP and IMV group (*p* = 0.035).

**Table 3:** Demographic and clinical characteristics according to ventilatory support in the epidemic season 2022–2023

	**SOT and HFNC** **(*N* = 241)**	**HCPAP and IVM** **(*N* = 53)**	***P*-value**
**Male, *N* (%)**	130 (53.9)	31 (58.5)	0.547
**Age, Days (IQR)**	66.1 (44.4–123.3)	58.2 (40.4–117.4)	0.437
**Preterm Birth, *N* (%)**	26 (10.8)	7 (13.2)	0.613
**Palivizumab, *N* (%)**	6 (2.5)	2 (3.8)	0.590
**Birth Weight, Grams (IQR)**	3330 (2960–3580)	3260 (2900–3630)	0.973
**Breastfeeding, *N* (%)**	184 (80.0)	43 (84.3)	0.479
**Siblings, *N* (%)**	152 (63.1)	38 (71.7)	0.234
**Comorbidities, *N* (%)**	13 (5.5)	5 (9.6)	0.261
**Los, Days (IQR)**	3.9 (2.9–5.9)	7.9 (4.9–10.9)	< 0.001
**Need for O2, *N* (%)**	186 (77.2)	53 (100.0)	< 0.001
**LOO, Days (IQR)**	3 (0.5–4)	6 (5–8)	< 0.00001
**Need for SOT, *N* (%)**	127 (52.7)	9 (16.9)	< 0.001
**Need for HFNC, *N* (%)**	88 (36.5)	52 (98.1)	< 0.001
**Coinfection, *N* (%)**	56 (23.2)	15 (28.3)	0.43
**RSV**	161 (68.5)	44 (83.0)	0.035

### 2018–2019 vs 2022–2023 season

According to the secondary objective, we compared the ongoing epidemic season with the last pre-pandemic season (2018–2019). Notably, we had almost the same number of patients in the two seasons: 294 admissions in the 2022–2023 season compared to 300 admissions in the 2018–2019 season.

**Fig. 2:**
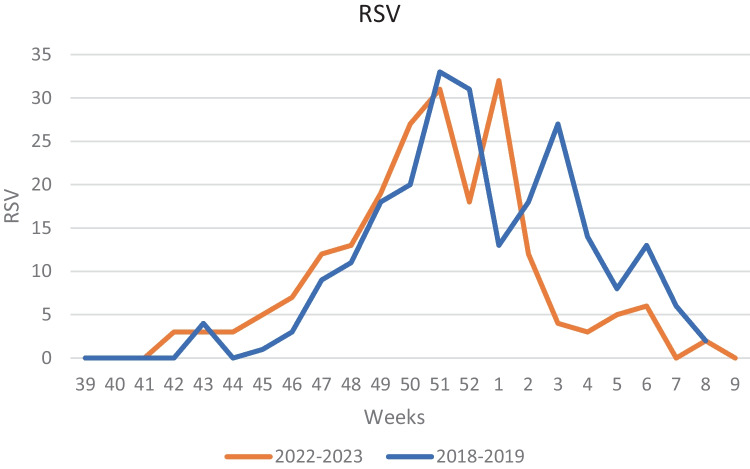
RSV detection in 2018–2019 and 2022–2023

**Fig. 3:**
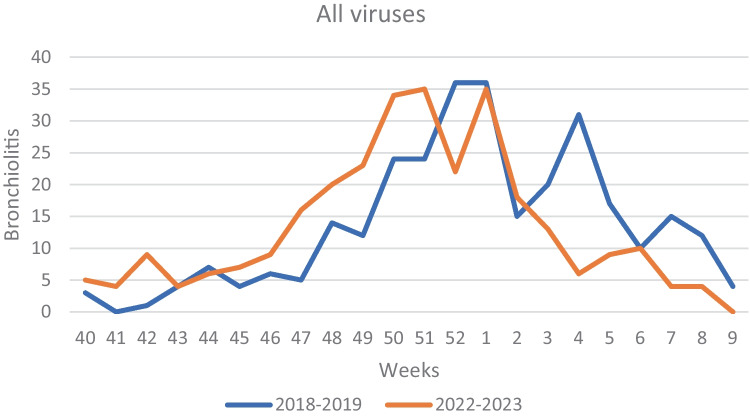
Respiratory virus detection in 2018–2019 and 2022–2023

**Fig. 4:**
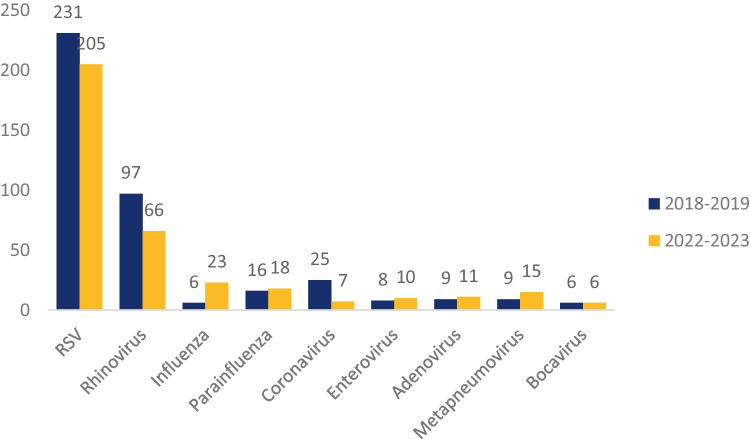
Viral detection in the two epidemic seasons

First RSV cases of the 2022–2023 season were detected at week 42, coinciding with the beginning of the epidemic season. The peak week was the 1st week of 2023 and RSV detections rapidly declined throughout January. The length of epidemic season was of 17 weeks, ending in week 6 of 2023. RSV epidemic season in 2018–2019 started at week 47, peaking in week 52 of 2019. The length of epidemic season was of 18 weeks, ending in week 12 of 2019 (Figs. 2 and 3). In Fig. 4, we reported the distribution of the viruses detected in the nasal swab in the two epidemic seasons analyzed. RSV results to be the major causative agents in both seasons.

Comparing the two seasons, we did not find any difference in terms of gender distribution, comorbidities, and prematurity. Age at admission was significantly higher in the current season (median age 2022–2023 65 days *vs* median age 2018–2019 58 days). Moreover, in the ongoing season, admitted patients had siblings in a smaller percentage (68.3% in 2022–2023 *vs* 76.4% in 2018–2019, *p* = 0,049). Clinical and demographic characteristics of the study population of the two epidemic seasons are shown in Table 1. LOS was significantly reduced in the 2022–2023 season, while the need for oxygen therapy was higher. Examining the type of respiratory support provided, a higher percentage of children in the ongoing season required HFNC (47.6% in 2022–2023 *vs* 40.3% in 2018–2019; *p* = 0.074), but differences were not statistically significant. Indeed, the need for HCPAP and for IMV was similar in the two epidemic seasons. Surprisingly, we found no differences based on breastfeeding.

## Discussion

In the present study, we examined epidemiology and characteristics of acute bronchiolitis in infants hospitalized in a single Italian pediatric hospital during the current epidemic season 2022–2023.

The median age of enrolled patients in our study was 66 days with a mild, not significant prevalence of male sex. An age less than 3 months at presentation is known to be a risk factor for severe bronchiolitis^1^. Other well-known risk factors for developing severe bronchiolitis are comorbidities and prematurity. In our series, 5.4% of patients presented comorbidities and 11.7% were born premature. Among them, only 8 (2.7% of total) received palivizumab. The remaining patients were not born premature or did not have severe comorbidities. In other words, most of patients hospitalized for bronchiolitis during the ongoing season were not eligible for the currently available RSV prophylaxis. This evidence suggests that there is a need for new therapeutic approaches allowing for an all-infants prevention strategy, including previously healthy children.

Most of our patients (81.3%) required a respiratory support. More specifically, 46.3% of them needed SOT, 47.6% HFNC, 17.7% HcPAP, and 8% IMV. Since there is no uniformity in HFNC and HcPAP treatment setting [23], we decided not to split our population into pediatric intensive care unit admission and ward admission. In fact, in our hospital, we provide HFNC in the ward and HcPAP in the pediatric emergency unit ward, as far as patient’s conditions permit. PICU in our hospital is generally reserved to severe respiratory distress at admission requiring HcPAP, HcPAP treatment failure, and IMV. Consequently, we decided to subdivide our patients into a group with less severe infection, who required only SOT and/or HFNC, and a group with more severe infection, who required HcPAP and/or IMV. The HcPAP and IMV group had higher rates of comorbidities and premature patients, but these data did not reach statistical significance.

According to literature [4], RSV is still the main etiological agent of bronchiolitis. During the ongoing epidemic season, it has been detected by nasal swabs in 71.2% of our patients, followed by rhinovirus (28.4%). In a literature review of 50 articles published between October 1999 and December 2017, RSV has been outlined as the most commonly detected virus (59.2%) as well [24]. In our cohort, viral coinfection was detected in 24.1% of patients, in agreement with previous studies that describe similar coinfection rates (between 25.8% and 34.7%) [25, 26].

Although it is confirmed that the number of viral agents detected positively correlates with days of oxygen therapy and length of stay, no significant differences emerged in terms of severity of clinical course between children with bronchiolitis due to a single and multiple virus.

However, patients requiring stronger ventilation support (HcPAP and IMV group) did not show higher coinfection rates. The clinical significance of coinfection remains unclear. Some studies reported increased severity of coinfection, but the impact of coinfection was not particularly obvious in other studies [25, 26].

Conversely, patients that tested positive for RSV were more likely to need longer hospital admission, required oxygen therapy more frequently, and had a higher length of oxygen therapy, corroborating previous investigations highlighting RSV as the most aggressive etiologic agent of bronchiolitis [27–29]. The burden of RSV-associated admission is confirmed by our previous study, proving that costs were significantly higher in children positive to RSV [28]. Noticeably, in our population, HcPAP and IMV patients had a significantly higher prevalence of RSV infection, confirming that one of the main drivers of disease severity is the etiological agent.

In addition to the acute medical and socioeconomic effects of RSV, there may be long-term consequences which contribute to a chronic disease burden. There is growing evidence showing an association between early-in-life RSV infection and later-life development of asthma and wheezing [30]. It is still unclear whether RSV infection is a causal factor, a marker of susceptibility to respiratory illness, or both^1^.

Hence again, it is important to broaden a cost-effective and efficient RSV-prevention strategy.

Comparing the ongoing epidemic season with the last pre-pandemic season (2018–2019), we found that the peak of hospitalization for bronchiolitis during 2022–2023 season occurred earlier.

We also showed that RSV season in 2022–2023 started earlier than usual. In fact, the epidemic season began more than 1 month in advance compared to the pre-pandemic season 2018–2019. Peak weeks were similar, as well as the length of the season: both are comparable to the median length of pre-pandemic seasons, which was of 16–18 weeks [8, 31], but 2022–2023 season ended earlier.

Based on historical RSV surveillance, RSV epidemic in Europe progresses rapidly after week 40 and the median start of the RSV season is in week 49 (i.e., beginning of December), ranging from week 41 to week 3 [31].

Due to the coronavirus disease of 2019 (COVID-19) pandemic, drastic changes in the epidemic curve of RSV have been reported. During the first year and a half of the pandemic, the number of cases of bronchiolitis considerably decreased worldwide because of the state-mandated public health measures to contain COVID-19 (lockdowns, school closures, social distancing, etc.) [12, 14]. After the wide-scale implementation of vaccination campaigns, easing of the restrictive measures against COVID-19 started worldwide in spring 2021; this led to an off-season resurgence of RSV infections in several countries around the world during last season (2021–2022) [16–18, 32].

Our data suggest that the current season still has atypical seasonality, starting anticipatedly and ending earlier, but compared to literature [7] and our 2018–2019 data, seasonality pattern may tend to realign to the pre-pandemic one.

Our data are in agreement with what is reported by several European countries [31].The ECDC report showed that the start of the 2022–2023 season was 5 weeks earlier than in the last three pre-COVID-19 seasons 2017/2018–2018/2019–2019/2020, but 14 weeks later than the 2021/2022 season, with differences between countries. Therefore, when comparing the last sentinel surveillance data with the pre-COVID-19 seasons, the average test positivity is at the levels of the 2018/2019 season.

RSV has been classified into two subtypes, A and B. These two major serotypes can simultaneously circulate during epidemic season, but, usually, one prevails over the other. Although some studies have shown that RSV-A is associated with increased disease severity, others have shown that either RSV-B is more severe or that the 2 subtypes have equivalent severity [6, 27, 33].

As illustrated above, in this 2022–2023 season, RSV-B was the primary circulating subtype, accounting for 85% of the subtyped samples. The RSV-B was the major causative agent also in the 2018–2019 season. The strong reduction of respiratory viruses’ circulation caused by COVID-19 restrictions may have altered the well-known alternating of RSV-subtypes circulation in our region. Monitoring the characterization of RSV and its circulating pattern could help in understanding the pathogenesis and the epidemiology of the infection; continued surveillance is required to determine the impact of the emergence of new genotypes on viral circulation, as well as on disease morbidity and mortality.

Even if the ongoing bronchiolitis season still demonstrates an unusual epidemiological trend, our data showed that disease severity was not different from the one we recorded before the COVID-19 appearance. In fact, even if our patients seemed to require oxygen therapy in a higher percentage in 2022–2023 compared to 2018–2019, length of stay resulted to be significantly lower in the ongoing epidemic season. Moreover, the percentage of patients requiring stronger ventilator support, such as HcPAP and IMV, was not different between the two seasons. In our opinion these results suggest a more aggressive attitude of clinicians rather than a more severe disease: as a matter of fact, more reliable indexes such as need for cPAP and intubation rate remained stable.

In literature, there is some concern that children that were not infected by respiratory viruses during the first 12 months of life because of pandemic might pay the immunological debt of missing viral infections^15^. However, we found that overall severity was not different from what expected from historical data, but admitted children were significantly older in the ongoing epidemic season. This may be explained by the lack of immunization due to the reduced circulation of respiratory viruses in the last years [34, 35].

Examining inpatients’ demographical data, in the ongoing epidemic season, children resulted to have siblings less frequently compared to 2018–2019, in line with other published data. Previous pandemics are already known to be associated with a decline in birth rates 9 months after their peaks. Also, COVID-19 pandemic influenced birth rates: the lack of information on the potential teratogenic effect, economic concerns, and maternal morbidity, and mortality associated with SARS-CoV-2 infection during pregnancy may have influenced the decision of couples to postpone pregnancies. The drop in livebirths in Italy has been estimated to be − 17.2% 9–10 months after the pandemic peak. This may have major consequences, especially in countries with an already low number of children per couple like Italy. Worsening demographical situation, our country did not show the birth rate rebound that other countries experienced 9–10 months after the end of lockdowns [36, 37].

In our cohort, the percentage of children who received palivizumab did not differ significantly between the prepandemic and current epidemic season and is still very low due to the strict administration rules. Consequently, RSV-related admissions do not differ significantly in 2018–2019 and 2022–2023 epidemic seasons.

Current strategies for the prevention of RSV disease in infants are limited to general preventive measures, such as handwashing and physical distancing, and passive immunity, for which there is only one approved therapy for high-risk infants, i.e., palivizumab. Despite extensive research efforts, there are no licensed vaccines to prevent RSV infection. However, multiple promising vaccine candidates are in clinical development for infants, pregnant women, and older adults. Potential vaccines currently being evaluated include live attenuated, gene-based vector, nucleic acid, chimeric, particle, and subunit vaccines [11, 38, 39]. RSV immunization during pregnancy has long been an attractive option since protection can be conferred throughout early infancy, when severe disease risk is especially high. This approach to RSV prevention would need to be complemented with other methods currently under development, as transplacentally acquired protection declines quickly postpartum [40]. An alternative strategy for RSV prevention in infants is the direct administration of mAbs with markedly improved characteristics compared with the first generation mAb, palivizumab. The greater and better knowledge of the structure and immunogenicity of RSV and its F protein has led to the development of latest generation monoclonal antibodies targeting epitopes placed on the Prefusion (Pre-F) antigen [38]. Among the new mAbs, the spearhead is nirsevimab. It offers protection for 5 months, enabling coverage of the entire RSV season with a single intramuscular dose. In a recent study [41], nirsevimab reduced medically attended RSV-associated LRTI by 80% and RSV hospitalization by 77% vs placebo in term and preterm infants. Recently, it has been approved by the European Medicines Agency [42]. The next step is to further review the complete data and efficacy of these strategies and to begin planning their implementation in real-life clinical settings. Important issues remain, such as understanding the optimal time during pregnancy for maternal vaccination and when to administer mAbs to infants in relation to RSV seasonality in the different parts of world. Additional issues include the need to better define the importance of providing cross protection for both RSV A and B strains and to monitor the potential emergence of RSV variants that could escape these preventive interventions [43]. Overall, the impact of RSV disease on health care systems and costs is concerning.

Reducing the global burden of RSV-related illness is considered a global health priority, and developing prevention strategies is a key priority for the WHO [44].

## Limits of the study

This study presents some limitations. It is a monocentric, monodepartmental, and retrospective study. In our hospital, indeed, many premature infants and newborns are hospitalized in the neonatal sub-intensive unit, and this could be a bias regarding the effective numbers of RSV bronchiolitis in our population. Therefore, we included in our study children aged less than 12 months, which may underestimate the variation in age of our patients in the ongoing season, given the hypothesis that children in their second year of life might have missed common respiratory diseases during the pandemic period.

## Conclusion

RSV is one of the major remaining common challenges in infectious diseases and a leading cause of hospitalization among children with less than 3 months. Our study showed that the expected 2022–2023 bronchiolitis season in Italy started and peaked earlier than the usual pre-pandemic seasons, but seasonality pattern may be realigning to the pre-pandemic one. This season was not more severe than the previous ones. Actually, RSV burden among the youngest patients underlines the need of protection for all infants. Understanding and updating the burden of childhood RSV disease are very important to support public health authorities and policy makers in the assessment of new preventive strategies against RSV disease.

## Data Availability

The datasets used and analyzed during the current study are available from the corresponding author on reasonable request.

## Abbreviations

HcPAP: Helmet continuous positive airway pressure
HFNC: High-flow nasal cannulas
IVM: Invasive mechanic ventilation
LOO: Length of oxygen supplementation
LOS: Length of hospitalization
mAB: Monoclonal antibody
PICU: Pediatric Intensive Care Unit
RSV: Respiratory syncytial virus
SOT: Standard oxygen therapy
